# A telephonic mindfulness-based intervention for persons with sickle cell disease: study protocol for a randomized controlled trial

**DOI:** 10.1186/s13063-017-1948-x

**Published:** 2017-05-15

**Authors:** Hants Williams, Susan Silva, Leigh Ann Simmons, Paula Tanabe

**Affiliations:** Duke University, Durham, NC USA

**Keywords:** Mindfulness, Mindfulness-based interventions, Sickle cell disease, Sickle cell, Chronic pain, Clinical trial, Telephonic, Pilot study

## Abstract

**Background:**

One of the most difficult symptoms for persons with sickle cell disease (SCD) to manage is chronic pain. Chronic pain impacts approximately one-third of persons with SCD and is associated with increased pain intensity, pain behavior, and frequency and duration of hospital visits. A promising category of nonpharmacological interventions for managing both physical and affective components of pain are mindfulness-based interventions (MBIs).

**Methods/design:**

The primary aim of this study is to conduct a randomized controlled study to evaluate the acceptability and feasibility, as well as to determine the preliminary efficacy, of a telephonic MBI for adults with SCD who have chronic pain. We will enroll 60 adult patients with SCD and chronic pain at an outpatient comprehensive SCD center in the southeastern United States. Patients will be randomized to either an MBI or a wait-listed control group. The MBI group will complete a six-session (60 minutes), telephonically delivered, group-based MBI program. The feasibility, acceptability, and efficacy of the MBI regarding pain catastrophizing will be assessed by administering questionnaires at baseline and weeks 1, 3, and 6. In addition, ten randomly selected MBI participants will complete semistructured interviews to help determine intervention acceptability.

**Discussion:**

In this study protocol, we report detailed methods of the randomized controlled trial. Findings of this study will be useful to determine the acceptability, feasibility, and efficacy of an MBI for persons with SCD and chronic pain.

**Trial registration:**

ClinicalTrials.gov identifier: NCT02394587. Registered on 9 February 2015.

**Electronic supplementary material:**

The online version of this article (doi:10.1186/s13063-017-1948-x) contains supplementary material, which is available to authorized users.

## Background

Sickle cell disease (SCD) is a genetic hematological disorder that affects more than 7 million people globally [1, 2]. It is estimated that 50% of adults with SCD experience pain on most days, with one-third experiencing chronic pain daily [3]. In addition to debilitating chronic pain [2, 4], persons with SCD experience acute pain episodes known as *vaso-occlusive crises* (VOCs). A VOC is caused by an accumulation of sickled red blood cells in the vasculature, resulting in damage (acute pain) to surrounding tissue areas [5]. In addition to pain caused by VOCs, it is common for persons with SCD to catastrophize pain (feelings of helplessness, pain rumination and magnification) and experience anxiety and depression [6, 7]. A promising category of nonpharmacological interventions for managing both physical and affective components of pain are mindfulness-based interventions (MBIs [8, 9]).

There is strong evidence to support the use of MBIs for persons with chronic pain [10, 11]. MBIs teach patients that sensing pain, even if it is intense or chronic, does not need to be fought, ignored, or suppressed or need to inhibit them from living a meaningful life or accomplishing their goals [12]. This approach challenges patients to decrease pain-related cognitive and emotional reactivity that can increase distress and exacerbate pain (e.g., pain catastrophizing) and to engage in active coping in the present moment [13]. The mindfulness approach is thus very different from other types of nonpharmacological therapies such as cognitive behavioral therapy (CBT). In CBT, a patient is taught to recognize and reframe negative thought patterns to change how they are feeling (e.g., analyzing thoughts; [14]), whereas in an MBI, the patient is taught not to reframe negative thoughts but to notice and accept thoughts (e.g., experiencing thoughts) and then redirect their focus to the present moment [15]. MBIs can thus be viewed as more of a naturalistic observation or participant observation (open monitoring) of thoughts vs. a purposeful reframing of thoughts as in CBT. For both approaches, multiple systematic and Cochrane reviews on their utility for pain and pain coping have been published, which largely support the use of both MBI and CBT approaches [16, 17].

Whereas MBIs have shown positive effects, the generalizability of these results for persons with SCD is unknown [18]. Some MBIs (e.g., mindfulness-based stress reduction [MBSR]) require eight weekly classes, homework assignments, and daily practice (30–45 minutes per day), which may be too much of a burden for persons with SCD and chronic pain. Second, missed clinic appointment rates for persons with SCD are reportedly as high as 46% [19], so it is unknown if persons with SCD are able to adhere to a weekly MBI schedule. Third, persons with SCD are not typically prescribed nonpharmacological interventions. Many persons with SCD are prescribed hydroxyurea and chronic opioids to manage their disease and have little exposure to additional therapeutic interventions, with the most common alternative therapy used by persons with SCD being prayer [20]. Last, the majority of current MBIs have been developed and tested with nonminority (Caucasian) sample populations, so there is uncertainty whether an MBI would be seen as acceptable, feasible, or effective in a predominately minority sample (e.g., African Americans with SCD). To our knowledge, no study to date has investigated the feasibility or acceptability of an abbreviated MBI delivered telephonically for persons with SCD and chronic pain [21]. In this paper, we report detailed methods of our clinical trial design.

### Research objectives

The overall goals of this pilot randomized controlled clinical trial (RCT) are to explore the feasibility and acceptability of an abbreviated six-session MBI that is targeted for persons with SCD and chronic pain and to obtain preliminary data on the efficacy of this intervention relative to a wait-listed control condition on pain catastrophizing and other pain-related outcomes. The specific aims are to (1) evaluate the feasibility and acceptability of the telephonic MBI designed to reduce pain-catastrophizing symptoms for adults with SCD and chronic pain and (2) determine preliminary efficacy of the MBI relative to the control condition on pain catastrophizing as well as pain interference and severity, depression, health-related quality of life (mental and physical health), and mindfulness

## Methods/design

### Overview

We are conducting a single-site pilot RCT comparing MBI and a wait-listed control condition with assessments at four time points (baseline, week 1, week 3, and week 6) in adult patients with SCD and chronic pain. Individuals who provide written informed consent will be enrolled and randomized to an MBI or control condition. The MBI will be conducted as a ten-person group teleconference call led by a certified MBI instructor. A 2:1 treatment allocation will be implemented in this initial pilot study to collect more data on the feasibility and acceptability of the MBI. Feasibility will include measures of enrollment, randomization, attendance, and intervention completion along with assessment completion. Acceptability will be assessed with semistructured interviews conducted with randomly selected MBI participants. The primary efficacy outcome will be pain catastrophizing total score.

#### Setting and sample

Patients will be recruited from an outpatient comprehensive SCD center in the southeastern United States. An interdisciplinary team of physicians, nurses, psychologists, and social workers run the center, providing care for approximately 600 adults with SCD. The target sample for this pilot study is 60 patients with SCD and chronic pain, with 40 patients randomly assigned to receive the MBI and 20 patients to the control condition. With a sample size of 60, the study does not have 80% power to detect a significant difference between the MBI and control conditions on the primary outcome of pain catastrophizing with a level of significance set a 0.05 (two-tailed). However, the focus of this pilot study is to estimate treatment effect sizes rather than to conduct statistical significance testing.

### Eligibility criteria

Patients meeting the following criteria will be eligible for inclusion: (1) self-reported diagnosis of SCD; (2) be age 18 years or older; (3) self-identified as having chronic, noncancer pain that has persisted on most days for more than 6 months and adversely affects function or well-being; (4) possess the ability to speak and read English; (5) have access to a landline or cell phone; and (6) have access to a compact disc (CD) or MP3 player. Patients will be excluded if they (1) previously participated in an MBI study (e.g., MBSR, mindfulness-based cognitive therapy or intervention) or (2) regular practitioners of mindfulness, including yoga.

### Recruitment

Recruitment letters will be mailed in waves of 50 to patients with a scheduled appointment at the outpatient center within 2 weeks of their appointment. This decision was made because there is no rigorous method to determine which patients are experiencing chronic pain. The recruitment letter will explain the study purpose, inclusion criteria (including definition of chronic pain), and participant involvement and provide contact information for the study principal investigator (PI). On the basis of previous research experience in the center, including poor response to recruitment letters, the recruitment letter will use institutional review board-approved opt-out language to notify patients that the PI will contact them via phone to describe the study, unless the patient informs the PI via phone or email that he or she does not want to be contacted. A period of 2 weeks will be provided prior to initiating phone calls after the mailing is sent to allow potential subjects time to contact the PI. Three attempts to contact the patient will be made before determining that the patient is not interested in participating. The same recruitment script used for in-person recruitment will be used to discuss the study with potential subjects by phone. Individuals who meet all inclusion criteria and verbally agree to participate will be mailed a consent form along with the study packet.

In-person recruitment will occur immediately before or after a patient’s clinic appointment. Prior to being approached by study personnel, patients will be prescreened by their healthcare provider and a study staff member to assess their general capacity to participate (e.g., ability to provide consent), current involvement in other research studies, and if they have already been mailed a recruitment letter. Patients already enrolled in a cognitive or behavioral intervention study will not be approached for recruitment, owing to the potential of carryover effects between interventions. A recruitment script will be used to discuss the study with potential participants, verify the presence of chronic pain per the study definition, and solicit participation. Individuals who meet all inclusion criteria and verbally agree to participate will then sign a consent form and receive a study packet containing a copy of the consent form, a set of headphones, a mindfulness practice CD, and four copies of assessments. For participants who have an email address, identical digital versions of the questionnaires will be provided through hyperlinks managed with REDCap electronic data capture tools [11].

### Randomization procedures

Patients who provide informed written consent and meet the participant selection criteria will be enrolled and randomized using a 2:1 treatment allocation to either the MBI or control condition. Because the MBI condition is group-based, randomization of participants will be divided into 4 cohorts of 15 participants to reduce the amount of idle time between informed consent and intervention delivery. After the first cohort of 15 participants provides informed consent, they will be randomly assigned either to receive the MBI (*n* = 10) or to a control condition (*n* = 5). The random number function in Excel 2007 software (Microsoft, Redmond, WA, USA) will be used to randomize patients to either the MBI (*n* = 40) or control (*n* = 20) condition using a permuted block randomization scheme to achieve a 2:1 allocation. Within each block size of 15, 10 patients will be randomized to receive the MBI and 5 will be randomly assigned to the wait-listed control condition.

Within 2 weeks of randomization, participants will begin the MBI or their time in the control condition. Following randomization and initiation of the first cohort of 15 patients, the next cohort of 15 patients will be recruited and randomized following the same protocol. This will continue until 60 participants are randomized in a total of 4 cohorts.

### Retention strategies and compensation

In an attempt to prevent participants from dropping out before the MBI starts, a proactive retention strategy will be used to retain participants who have been randomized but have not yet participated in the MBI. Participants will receive weekly contact via telephone and email and will be provided with information regarding how many more participants are needed before the intervention will begin. After beginning the MBI program, participants randomized to the MBI will receive either a telephone call, a text message, or an email (their choice) on the morning of each mindfulness session and again 1 h before each session. In addition, all participants will receive weekly and biweekly contact from study personnel to remind them of their study participation, upcoming due dates for assessments, and when compensation checks have been mailed to their home address.

Three monetary payments will be provided to help increase retention and compensate participants for their time: (1) $10.00 after baseline assessments are received and/or participation in MBI session 1 has occurred, (2) $20.00 after receiving measurements at the end of week 3 and/or participation in MBI session 3, and (3) $30.00 at the conclusion of the study upon receiving the final measurements and/or participation in MBI session 6. All subjects in the in MBI and control groups will receive payments upon return of each assessment according to this schedule. In addition to providing monetary compensation, the MBI program will be provided at no cost to subjects, a value of over $400.00.

### Description of intervention arms

#### Mindfulness-based intervention condition

Participants randomized to the MBI condition will receive a telephonically delivered MBI program. The MBI adapts all core elements from John Kabat-Zinn’s original MBSR program [23] and targets common symptoms, emotions, and stressors related to chronic pain experienced by persons with SCD. We developed the telephonic MBI through an informal process that involved iterative feedback from patients, clinical experts in SCD and pain management, social workers, psychologists, and mindfulness clinicians. Through this process, relevant topics and skills were selected from the original MBSR program to adapt for each MBI session.

On the basis of needs described, and in line with adapting specific elements of MBSR, the original MBSR content was simplified and compressed into six 60-minute telephonic group sessions that teach (1) breath awareness (focus on breathing and observing thoughts without fighting or following them, weeks 1–6), (2) body scan (promoting mindfulness of sensations in different parts of the body, weeks 2–6), (3) walking meditation (walking as form of meditation, weeks 3–6), (4) loving-kindness (projection of friendliness and kindness toward oneself and others, weeks 4–6), and (5) choiceless awareness (awareness of all sensations with equal interest, weeks 5–6). Each of these practices, all of which are part of the original MBSR program, is focused on cultivation and practice of mindfulness techniques. Breath awareness, body scan, loving-kindness, walking meditation, and choiceless awareness promote and foster “open monitoring” and intentional observation of thoughts, feelings, and bodily sensations [13]. With guidance from the mindfulness instructor, participants will be presented with various examples of how each exercise can be infused with everyday tasks (e.g., doing the dishes, brushing teeth).

Currently, there is much debate on the mechanisms of how each of these practices may elicit changes for pain-related outcomes [15], but because the MBI contains components of exposure therapy, the walking meditation and body scan exercises may contribute to decreased pain-related disability by reducing fear of movement and fear of pain [24]. Researchers are actively engaged in trying to better understand the mechanisms of mindfulness and how mindfulness practices may specifically help those with pain [25–28].

Table 1 summarizes the intervention that will be used in this study and compares it with typical MBSR programs. The first 10 minutes of each session will be used to review previous material, followed by approximately 20 minutes of instruction and overview of a new mindfulness exercise and 15 minutes of mindfulness practice, with the remaining time used for questions. Because each session will be conducted as a group teleconference call, the size of each MBI session is limited to a maximum of ten patients (plus the mindfulness instructor) to minimize caller interruptions and disruptions.

**Table 1:** Comparison between typical mindfulness-based stress reduction program and telephonic mindfulness-based intervention for persons with sickle cell disease

Week	MBSR	Telephonic MBI
1	Body scan	Mindful breathing
2	Breath awareness	Body scan
3	Sitting meditation, individual yoga	Loving-kindness
4	Stress coping	Mindful eating
5	Communication styles	Sensory awareness
6	Yoga, sitting meditation	Overview, conclusion
7	Loving-kindness	N/A
Weekend Retreat	Silent retreat	N/A
8	Wrap-up	N/A

*MBSR* Mindfulness-based stress reduction, *MBI* Mindfulness-based intervention, *N/A* Not applicable

This table provides a comparison of the standard 8-week MBSR program and the telephonic MBI that will be tested

Our decisions to reduce the number of minutes per session and remotely deliver the MBI are supported by the MBI literature. Prior studies have found time commitment to be a common barrier to recruitment and MBI completion [29, 30], as well as a nonsignificant correlation between the number of in-class hours and effect size of an MBI [31]. Therefore, first, the number of minutes per session has been reduced to 60, a length we believe is more amenable to the subject’s other obligations (e.g., work and family activities), and the number of weekly sessions has been reduced from eight to six. Second, it is difficult for some persons with SCD to find transportation to and from the clinic; therefore, an intervention that is easily accessible by telephone may increase the likelihood of recruitment, retention, and program completion. The literature supports the delivery of MBIs by telephone, with telephone vs. in-person interventions producing comparable outcomes [32–34]. Therefore, the six MBI sessions will be delivered telephonically instead of in person. In addition, the 2011 Pew Internet and American Life Project reported that approximately 87% of African Americans have cell phones and that 73% of individuals who make less than $10,000 per year have phones [35], providing support for the majority of persons with SCD being likely to have a phone.

The same MBI instructor will deliver all MBI sessions. The instructor is a healthcare professional and graduate of the University of Massachusetts Center for Mindfulness professional training program. In total, the instructor has more than 10 years of instructional experience in mindfulness, 2 years of experience in teaching telephone-based MBSR, and experience in leading groups for research studies.

Last, each 60-minute MBI session will require patients to call a toll-free 1-800 telephone number. The toll-free number does not require an access or login code; can accommodate up to 100 callers simultaneously; and can be accessed by telephone, Internet, and mobile app (iOS and Android only). The conference service used will be UberConference (Dialpad, San Francisco, CA, USA).

#### Wait-listed control condition

Patients randomized to the control condition will not receive the MBI but will receive treatment as usual. Treatment as usual consists of standard medication management as prescribed by the subject’s hematologist, primary care physician, psychologist, and/or any other medical professionals overseeing treatment.

**Fig. 1:**
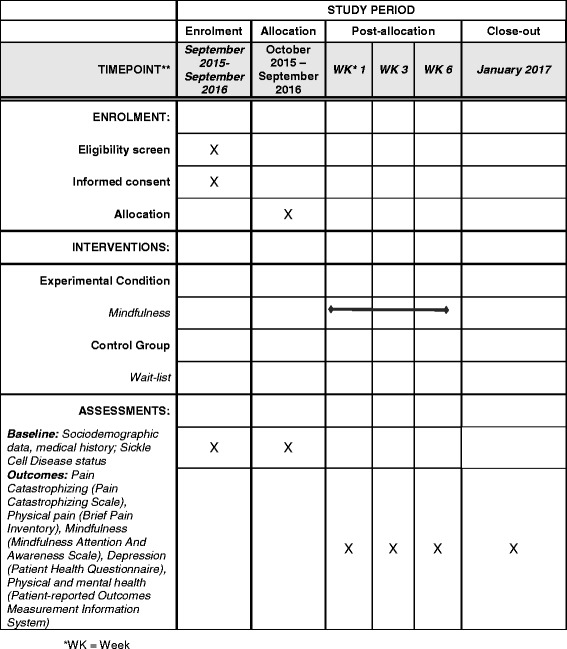
Schedule of enrollment, interventions, and assessments (formatted on the basis of the Standard Protocol Items: Recommendations for Interventional Trials [SPIRIT] 2013 template)

Wait-listed patients will be offered the opportunity to cross over to the MBI condition once patients in the intervention arm complete six sessions. Patients who cross over from the wait-list to the MBI will participate in the same protocol as patients originally randomized to the MBI condition. The schedule of enrollment, interventions, and assessments of this study is shown in Fig. 1 and the Standard Protocol Items: Recommendations for Interventional Trials (SPIRIT) checklist (*see* Additional file 1).

### Assessments

#### Demographic assessment

Sociodemographic data and clinical characteristics will be self-reported by participants and collected only at study enrollment (baseline). Participants will be asked to report their sex, age, race, genotype, socioeconomic status, educational history, hospital use (e.g., self-reported number of emergency department visits and hospital admissions over the last 2 years), and disease-related complications (e.g., stroke, acute chest syndrome, VOCs). These items have been used to assess sociodemographic and clinical characteristics of persons with SCD in prior studies [36, 37].

### Feasibility

The first aim of this study is to determine feasibility and acceptability. Measures of feasibility are (1) enrollment (number of participants consented and enrolled) and (2) randomization (percentage of patients enrolled who are randomized). Participant-level measures of feasibility include (1) attendance (percentage of sessions attended by each MBI participant), (2) intervention completion (percentage of total intervention sessions attended by each MBI participant), and (3) assessment completion (percentage of completed patient-reported assessments at each time point). Failure to complete measures within 2 weeks of recruitment (baseline) or MBI sessions 1, 3, and 6 results in missing data for that time point.

### Acceptability

Acceptability will be assessed qualitatively. Semistructured interviews will be performed with ten randomly selected participants from the MBI condition within 2 weeks of the final MBI session by telephone to assess acceptability and help interpret quantitative findings related to the exploratory aim [38]. Each MBI participant who completes at least one MBI session will be randomly assigned a number using the random number function in Excel 2007 and then ordered in an ascending fashion. The participant with the lowest number will be contacted first, followed by the participant with the next lowest number, and so forth. Two to three participants will be selected from among each of the four cohorts. Each interview will be conducted by one study staff member who is familiar with the content of the MBI. The interview will consist of the questions listed in Table 2.

**Table 2:** Semistructured postinterview questions

Q1	Tell me about how easy or difficult it was for you to complete the weekly telephone-based MBI sessions?
Q2	What impact, if any, do you feel the MBI program had on (a) your perceptions on chronic pain, and (b) your quality of life?
Q3	What did you find most useful about the MBI program?
Q4	What did you find least useful about the MBI program?
Q5	What would you want more of in the MBI program?
Q6	What would you eliminate or have less of in MBI program?
Q7	Tell me about how easy or difficult it was for you to practice mindfulness, and complete the homework assignments?
Q8	Do you intend to continue using this MBI?
Q9	Would you recommend a MBI to friends with SCD who also experience chronic pain?
Q10	Is there anything else that you would like me to know about your experience?

*MBI* Mindfulness-based intervention, *SCD* Sickle cell disease

This table provides a list of questions that will be asked of study participants who are randomized to the experimental condition, and it will be administered at the end of the 6-week MBI

Because we were unable to find any literature on the subjective experiences of persons with SCD and chronic pain who participated in a mindfulness intervention, the guiding questions listed in Table 2 will be used to roughly structure conversations, allowing digression into other topics brought up by the participants. Interviews will be digitally recorded, and we anticipate that they will last between 30 and 60 minutes.

### Efficacy

To address aim 2, five valid and reliable patient-reported questionnaires will be administered to determine the effects of MBI relative to the control condition on pain catastrophizing, as well as on pain interference and severity, depression, health-related quality of life (mental and physical health), and mindfulness (Table 3). Each questionnaire will be administered at four time points: week 0 (baseline, prior to initiation of treatment) and weeks 1, 3, and 6. Assessments at weeks 1, 3, and 6 correspond to MBI session 1, session 3, and session 6, respectively.

**Table 3:** Instruments and schedule of assessments

Instrument	Abbreviation	Domain	Scale/subscale reliability (Cronbach’s α)	Interpretation^a^	Baseline	Week 1	Week 3	Week 6
Demographic Questionnaire	DEMO	Sociodemographic and clinical characteristics	N/A	N/A	X			
Pain Catastrophizing Scale	PCS	Pain catastrophizing^b^	Total score 0.75 Rumination 0.87 Magnification 0.60 Helplessness 0.79	Low	X	X	X	X
Brief Pain Inventory	BPI	Pain interference and severity	Interference 0.89–0.92 Severity 0.80–0.87	Low	X	X	X	X
Patient Health Questionnaire–9 items	PHQ-9	Depression	Total score 0.87	Low	X	X	X	X
Patient-Reported Outcome Measurement Information System	PROMIS	Physical and psychological health	Mental and physical 0.73–0.96	High	X	X	X	X
Mindful Awareness Attention Scale	MAAS	Mindfulness	Total score 0.87	High	X	X	X	X

*N/A* Not applicable

This table provides the list of questionnaires that will be administered to study participants. Additionally, this table provides details related to when each questionnaire will be administered; the questionnaire’s abbreviation, domain, and psychometrics; and how the questionnaire should be interpreted

^a^Scores representing “improved” functional health status

^b^Primary outcome

Participants will have the option of completing each questionnaire on paper or via an online form. Participants who opt for paper forms will be provided with a packet of questionnaire copies with return envelopes and stamps. Participants who opt for online forms will provide an email address and be emailed hyperlinks that contain exact copies of the paper forms managed by a REDCap database [22]. The order and structure of each measurement will be identical in the paper and online versions. Each assessment packet includes the five questionnaires described in the subsections below. *See* Table 3 for a summary of instruments and schedule of assessments.

#### Pain Catastrophizing Scale

The Pain Catastrophizing Scale (PCS) will be used to assess pain catastrophizing. The PCS contains 13 items rated on a 5-point scale from 0 (not at all) to 4 (all the time). The total score for the PCS ranges between 0 and 52, with a higher score demonstrating more severe catastrophizing. In addition, the PCS contains three subscales: helplessness (six items), rumination (four items), and magnification (three items) [39]. The PCS was selected as the primary variable of interest in addressing intervention efficacy because it has been studied extensively across many different chronic pain populations and is commonly used as a key outcome in determining the success of interventions that target chronic pain [40, 41]. A total PCS score of 30 represents a clinically relevant level of catastrophizing and corresponds to the 75th percentile of the distribution of PCS scores in clinical samples of chronic pain patients [42]. The PCS total score was the primary outcome.

#### Brief Pain Inventory

The Brief Pain Inventory (BPI) will be used to assess physical pain. As one of the standard psychometric tools for clinical trials of pain [43, 44], the BPI provides two subscales: pain interference and pain severity. Pain interference (seven items) and pain severity items (five items) are rated on a 0–10 scale, with 10 indicating complete interference or worst possible pain severity. There are no clinical cutoff scores for the BPI, but “worst pain” or the arithmetic mean of the four severity items can be used as measures of pain severity, and the arithmetic mean of the seven interference items can be used as a measure of pain interference.

#### Patient Health Questionnaire–9 items

The Patient Health Questionnaire–9 items (PHQ-9) will be used to assess depression. The PHQ-9 is a multipurpose instrument used for screening, diagnosing, monitoring, and measuring the severity of depression [45]. Consisting of nine items on a Likert scale (0 = not at all, 3 = nearly every day), the measurement provides a severity score for depression (range 0–27) of minimal symptoms (5–9), minor depression (10–14), moderate depression (15–19), or severe depression (20–27).

#### Patient-Reported Outcomes Measurement Information System

The Patient-Reported Outcomes Measurement Information System (PROMIS) [47] global short form will be used to assess physical and mental health. The PROMIS global short form is a ten-item instrument that represents multiple domains developed by the National Institutes of Health. The tool has a total of ten items with a Likert scale (0–5) and generates a global physical health score and a global mental health score [46]. Currently, there are no clinical cutoff scores for chronic pain for PROMIS, but they are currently being developed [47].

#### Mindful Attention Awareness Scale

Mindful Attention Awareness Scale (MAAS) will be used to assess mindfulness. The MAAS measures present moment awareness, interpersonal communication, thoughts, emotions, and physical states. Consisting of 15 items on a Likert scale (1 = almost always to 6 = almost never), the measurement provides a single total score of mindfulness, with a higher score indicating a higher level of mindfulness.

### Analytic approaches

Atlas ti 7™ software (Scientific Software Development, Berlin, Germany) will be used to analyze qualitative data, and SAS version 9.3™ software (SAS Institute, Cary, NC, USA) will be used to conduct statistical procedures for quantitative data and to estimate effect sizes for the efficacy outcomes. When statistical significance testing is conducted, nondirectional tests will be performed, with the level of significance set at 0.05. As noted earlier, the focus of this pilot study is to examine the direction and magnitude of effect of the MBI on primary and secondary outcomes rather than to conduct statistical significance testing.

### Sample characteristics

Descriptive statistics will be used to summarize sociodemographic and clinical characteristics. Nonparametric Wilcoxon two-sample tests will be used to test for differences in continuous sociodemographic and clinical variables between conditions. Chi-square tests will be performed to test for differences in categorical sociodemographic and clinical variables between conditions. A sensitivity analysis will be performed to compare sociodemographic and clinical characteristics between randomized participants who complete at least one assessment at any time point with those who do not complete any outcome assessments (observable cases = no or yes).

### Feasibility

Number and percent will be used to summarize enrollment, randomization, assessment completion, intervention completion, and MBI session attendance. Additionally, the sociodemographic and clinical characteristics of those observable subjects in the MBI condition who complete four or more intervention sessions will be compared with those who complete less than four.

### Acceptability

Acceptability will be determined by qualitative analysis of semistructured interviews using an inductive data-driven approach. This will allow for extraction of core themes, as recommended by, for example, Hsieh and Shannon [48], for cases when theory or research literature is limited. Qualitative content analysis is selected because it is a widely and successfully used method for qualitative data analysis [49]. A multistage analytic strategy has been developed for qualitative analysis. In step 1, digital recordings will be transcribed verbatim. In step 2, transcripts will be imported into Atlas.ti software and then read and reread by three study personnel familiar with the content of the MBI, and text passages that appear to be relevant with regard to the research questions will be extracted and coded (i.e., labeled as a term preferably close to the text passage itself). Further relevant text passages will be subthemed under an existing term or assigned a new term if they do not fit into an existing category. In this style, the first two transcripts will be coded. In step 3, the emerging categories will be reviewed and critiqued by the three study personnel in a group discussion and revised. In step 4, the first two transcripts will be recoded on the basis of the discussion, and the next two transcripts will be coded on the basis of adjustments to the coding scheme determined by the three coders. In step 5, the three study personnel will reassemble to discuss the first four interviews and any new emergent codes. In step 6, the remaining five interviews will be coded.

### Efficacy

Descriptive statistics will be used to summarize the five measures at baseline and weeks 1, 3, and 6 for randomized participants with at least one outcome assessment completed (observable cases). Tables for unadjusted mean scores for each condition by time will be presented. Random coefficients regression models, a type of hierarchical mixed-effects model for longitudinal data, will be used to evaluate the trajectory of change in pain catastrophizing total scores and other pain-related outcomes. Nonlinear temporal patterns will be fitted as needed. The models will be used to examine the pattern of change in each outcome over time and estimate effect sizes.

## Discussion

This pilot will provide preliminary evidence regarding the feasibility, acceptability, and efficacy of a telephonic MBI for persons with SCD and chronic pain. Considering how difficult it is to manage SCD, pain in particular, this trial will provide valuable insight into whether a telephonic MBI may help improve the care of persons with chronic pain and SCD. We believe the results of this trial will provide significant new insights for the development of future MBIs for persons with SCD and provide additional information regarding the efficacy of a remote intervention for managing a chronic disease.

### Trial status

Participants are no longer being recruited.

## Additional file

Additional file 1: Standard Protocol Items: Recommendations for Interventional Trials (SPIRIT) 2013 checklist: recommended items to address in a clinical trial protocol and related documents. (DOC 121 kb)

## Abbreviations

BPI: Brief Pain Inventory
CBT: Cognitive behavioral therapy
CD: Compact disc
DEMO: Demographic Questionnaire
MAAS: Mindfulness Awareness Attention Scale
MBI: Mindfulness-based intervention
MBSR: Mindfulness-based stress reduction
PCS: Pain Catastrophizing Scale
PHQ-9: Patient Health Questionnaire–9 items
PI: Principal investigator
PROMIS: Patient-Reported Outcome Measurement System
RCT: Randomized controlled clinical trial
SCD: Sickle cell disease
SPIRIT: Standard Protocol Items: Recommendations for Interventional Trials
VOC: Vaso-occlusive crisis
